# CLINICAL FACTORS ASSOCIATED WITH THE QUALITY OF INTERACTIONS BETWEEN STAFF AND HOSPITALIZED PERSONS WITH DEMENTIA

**DOI:** 10.1093/geroni/igad104.0304

**Published:** 2023-12-21

**Authors:** Anju Paudel

**Affiliations:** Penn State Ross and Carol Nese College of Nursing, State College, Pennsylvania, United States

## Abstract

The quality of care greatly depends on the quality of interactions between staff and hospitalized older adults with dementia which in turn can be influenced by many factors. This paper examines the clinical factors associated with the quality of interactions between staff and hospitalized older adults with dementia. Following the examination of bivariate associations, we conducted multiple linear regression in a sample of 140 hospitalized older patients with dementia who participated in the final cohort of Fam-FFC. On average, the participants (male = 46.1%, female = 52.9%) were 81.43 years old (SD = 8.29) and had positive interactions with staff. Accounting for 17.5% of the variance in the model, non-pharmacological interventions (b= 0.181; p<.001) and pain (b= -0.201; p<.01) were significantly associated with the quality of staff-patient interactions. To optimize care of hospitalized patients with dementia, staff should be encouraged to use non-pharmacological interventions and prioritize pain management in patients.

